# The Light Wavelength Affects the Ontogeny of Clock Gene Expression and Activity Rhythms in Zebrafish Larvae

**DOI:** 10.1371/journal.pone.0132235

**Published:** 2015-07-06

**Authors:** Viviana Di Rosa, Elena Frigato, José F. López-Olmeda, Francisco J. Sánchez-Vázquez, Cristiano Bertolucci

**Affiliations:** 1 Department of Physiology, Faculty of Biology, Regional Campus of International Excellence “Campus Mare Nostrum”, University of Murcia, Murcia Spain; 2 Department of Life Sciences and Biotechnology, University of Ferrara, Ferrara, Italy; Karlsruhe Institute of Technology, GERMANY

## Abstract

Light plays a key role in synchronizing rhythms and setting the phase of early development. However, to date, little is known about the impact of light wavelengths during the ontogeny of the molecular clock and the behavioural rhythmicity. The aim of this research was to determine the effect of light of different wavelengths (white, blue and red) on the onset of locomotor activity and clock gene (*per1b*, *per2*, *clock1*, *bmal1* and *dbp*) expression rhythms. For this purpose, 4 groups of zebrafish embryo/larvae were raised from 0 to 7 days post-fertilization (dpf) under the following lighting conditions: three groups maintained under light:dark (LD) cycles with white (full visible spectrum, LDW), blue (LDB), or red light (LDR), and one group raised under constant darkness (DD). The results showed that lighting conditions influenced activity rhythms. Larvae were arrhythmic under DD, while under LD cycles they developed wavelength-dependent daily activity rhythms which appeared earlier under LDB (4 dpf) than under LDW or LDR (5 dpf). The results also revealed that development and lighting conditions influenced clock gene expression. While *clock1* rhythmic expression appeared in all lighting conditions at 7 dpf, *per1b*, *per2* and *dbp* showed daily variations already at 3 dpf. Curiously, *bmal1* showed consistent rhythmic expression from embryonic stage (0 dpf). Summarizing, the data revealed that daily rhythms appeared earlier in the larvae reared under LDB than in those reared under LDW and LDR. These results emphasize the importance of lighting conditions and wavelengths during early development for the ontogeny of daily rhythms of gene expression and how these rhythms are reflected on the behavioural rhythmicity of zebrafish larvae.

## Introduction

Circadian rhythms are regulated by an endogenous system of circadian oscillators that act in harmony with the environmental cycles. They represent an adaptive advantage that allows organisms to predict and anticipate cyclic environmental changes [1, 2]. Light and temperature are the predominant signals for the entrainment of circadian oscillators [1]. Light acts through changes in intensity (day/night variations) and photoperiod (seasonal variations), while temperature influences biological rhythms through natural oscillations of the thermo-cycle (both daily and seasonal) [3]. In the aquatic environment, light has an additional feature since the spectral composition of incident light changes with depth: longer wavelengths (reddish) are absorbed rapidly, while shorter wavelengths (bluish) become predominant [4, 5]. Thus, in fish, photic sensitivity depends on adaptation of their photoreceptor system (lateral eyes, pineal gland, deep brain and dermal photoreceptors) to the different properties of the light in the water column, which change depending on factors such as chemical composition, organic substances and depth [5, 6]. Zebrafish is a small cyprinid teleost fish, native of the Ganges River in eastern India, traditionally considered a diurnal species in both its adult and larval stages [7–9]. It has become a common model species used to investigate the vertebrate circadian clock, embryology and developmental biology [10–12], and it is an ideal candidate model for studying the effect of light on the development and early emergence of light-responsive structures [13–16].

Several studies have investigated the effect of light on the locomotor activity of zebrafish adults and larvae, focusing on light cycles and daylengths [7, 8, 16–19]. Depending on water temperature, zebrafish larvae hatch at 2–3 day post-fertilization (dpf) and remain generally inactive prior to inflation of the swim bladder and the start of feeding (4–5 dpf). Adult and larvae zebrafish are mostly active (>65%) during the light phase and circadian rhythmicity persists in constant darkness [7, 8, 16]. Interestingly, previous investigation pointed out the importance of light during early development because only 20% of larvae reared under constant darkness showed circadian rhythmicity, further stressing the importance of entraining signals (i.e. light) to initiate circadian rhythmicity, which is regulated by a pacemaker sensitive from 2 dpf [17]. Furthermore, a recent paper on light composition also showed that different light spectra have different effects on growth performance in zebrafish [20]. Similar investigations in marine fish, such as sole (*Solea senegalensis*) and sea bass (*Dicentrarchus labrax*) confirmed the importance of light characteristics for embryo development, hatching rhythms and larval growth [21, 22].

The circadian clock mechanism that regulates the rhythmicity in vertebrates consists of interacting positive and negative transcriptional/translational feedback loops. Positive elements such as CLOCK and BMAL bind to E-box elements located in the regulatory regions of negative elements (*per*s and *cry*s). CRY and PER proteins down-regulate their own expression by inhibiting CLOCK-BMAL [23], allowing the feedback loop to exist. The transduction of circadian information is achieved by rhythmic activation of clock-controlled output genes that regulate downstream processes [24]. For instance, DBP is a D-box binding protein whose rhythmic expression is driven by CLOCK-BMAL through an E-Box-mediated activation [25, 26]. These transcription factors controlled by the clock confer circadian expression on downstream genes, modulating various physiological processes [27]. In zebrafish, the existence of multiple forms of the key clock genes *cry*, *per*, *clock* and *bmal* has been reported [28, 29]. *Per1* is a clock-controlled gene that is present with two homologues (*per1a* and *per1b*), whereas *per2* is a light-driven gene necessary for the ontogeny of the clock [30].

Different investigations have described the role of LD cycles on the expression of different light-responsive genes involved in the molecular clock [31–36]. Dekens and Whitmore (2008) observed the light-independent initiation of zygotic *per1* transcription in the first day of development, whose oscillations were asynchronous [15]. Moreover, during the first 3 days of development, *clock* and *bmal1* were not rhythmic, and the onset of their rhythmic expression coincided with the appearance of several circadian clock output processes, such as locomotor activity and DNA replication [15, 16, 37]. However, to date, the effects of different light wavelengths on the ontogeny of molecular clock genes remain unknown.

The objectives of the present research were to investigate the influence of 12:12 light-dark (LD) cycles with a different light spectrum (white, LDW; blue, LDB; red, LDR) on the ontogeny of clock gene and behavioural rhythms in zebrafish. For this purpose, we first looked at the locomotor activity, a well-known circadian behavioural output, on larvae reared under different light conditions during the first 7 days of life, in order to ascertain whether rhythmic patterns depend on photic conditions. Next, we focused on a subset of core-clock (*per1b*, *clock1*, *bmal1*), light-regulated (*per2*), and clock-output (*dbp*) genes to study how changes in the lighting conditions might alter the onset of daily and circadian gene expression.

## Materials and Methods

### Ethics Statement

The present research was carried out in the Chronobiology laboratories of the University of Murcia (Spain) and of the University of Ferrara (Italy). All husbandry and experimental procedures complied with European Legislation for the Protection of Animals used for Scientific Purposes (Directive 2010/63/EU). The experimental protocol was previously authorized by the Spanish National Committee on Animal Welfare (Law 32/2007) and the Bioethical Committee of the University of Murcia (Spain) and by the University of Ferrara Institutional Animal Care and Use Committee and the Italian Ministry of Health.

### Animal rearing

Wild-type adult fish were obtained from commercial provider (Alimar Pets S.L., Murcia, Spain) and housed for 1 year in 9 L glass aquaria (1 fish l^-1^) according to standard method [38]. The reproductive fishes were fed 2 and 6 hours after lights on with dry food (Tropical fish flakes; PRODAC, Italy). For spontaneous spawning, the sexually mature fish were separated in groups of 5 fish (3 females and 2 males) and transferred into a 2.5 L breeding net cage during the afternoon. Spawning took place the next morning, approximately 2 hours after lights on. Eggs were collected, pooled and distributed into 85×10 mm plastic Petri dishes with cover (20 eggs per Petri dish) filled with embryo medium [38]. The Petri dishes were placed to float in a 12 liters aquarium at 27°C.

### Experimental procedure

The experimental groups were exposed to four different lighting conditions: LDW (white), LDB (blue), LDR (red) and DD (constant darkness). The light-dark (LD) cycle was of 12 hours light and 12 hours darkness. Illumination was provided by means of neutral red, blue, and white LED light lamps (Superlight Technology Co. Ltd., China). Irradiance was measured with a spectro-radiometer (FieldSpec ASD, Colorado, USA) set at 1.62 E+18 photons m^-2^ s^-1^. The λ_max_ of the red and blue LED light lamps were 639 nm and 465 nm, respectively. The temperature was held constant (27°C) by means of water heaters (50 W, Sera GmbH, Germany) and recorded every 10 minutes with data loggers (Hobo Pendant, Onset Computer Corporation, Massachusetts, USA).

### Behavioural recording

The embryos were collected immediately after spawning, incubated in 12-well clear bottom plastic plates filled with 5 ml of embryo medium and exposed to a specific light condition. Each multi-well plate was placed on the water surface of a 10 L aquarium. The plate was fixed on the base of the aquarium to avoid any change of position during the recording. The larvae were fed at 5 dpf and the embryo medium was partially changed every 2–3 days. Swimming activity patterns were recorded from 2 to 7 dpf, for each light condition. Larvae were recorded by means of a webcam adapted for infrared recording by removing the UV filter in front of the lens placed on the top of the aquarium and connected to a computer. An infrared LED (monocolor diode, model L-53F3BT, 5 mm) covered with a blurred white panel was placed under the aquarium to permit the video recording during the dark phase of experiment. These IR lamps emitted at 940 nm, which is not detected by zebrafish [39]. Two specialized software packages, Multiviewer and FishTracker (Computer System Department, University of Murcia), were used. The Multiviewer allowed simultaneous webcam recording. Every minute, 60 images (1 frame/s) were stored. The FishTracker quantified the larvae movements and has already been validated in sea bream [40] and zebrafish [41].

### Molecular analysis

Embryos and larvae were maintained under different light conditions in Petri dishes and sampled at five different time points (ZT/CT 3, ZT/CT 9, ZT/CT 15, ZT/CT 21; ZT 0 = lights on, ZT 12 = lights off), during day 0, 3 and 7 post fertilization (dpf). For each ZT/CT, 20 embryos at 0 dpf and 10 larvae at 3 and 7 dpf were sampled and pooled. Four pooled samples per ZT/CT were collected (n = 4). Total RNA was isolated from zebrafish embryos and larvae using Trizol reagent (Invitrogen, Carlsbad, CA, USA) following the manufacturer’s instructions. The amount, quality and composition of isolated RNA were analysed by BioSpec-nano (Shimadzu, Kyoto, Japan). One microgram of total RNA was incubated with DNase I (Invitrogen) at room temperature for 30 min and then at 85°C for 15 min to inactivate the enzyme. DNase-treated RNA was used to perform cDNA synthesis in a final volume of 20 μl, using iScript cDNA Synthesis Kit (Biorad, Milan, Italy). The reaction was performed at 42°C for 30 min, followed by an inactivation step of 5 min at 85°C. Three microliters of 1:10 diluted first-strand cDNA was PCR amplified with a Chromo4 Real-Time PCR Detection System (Bio-Rad, Milan, Italy) using SsoFast EvaGreen Supermix (Bio-Rad Laboratories, Hercules, CA, USA). Thermal cycling conditions were as follows: 3 min denaturation at 95°C, followed by 40 cycles of a 5 s denaturation step at 95°C and an annealing-elongation step for 20 s at 60°C. After amplification, a melting curve analysis to confirm the specificity of the amplicon was performed from 60 to 95°C, with increments of 0.5°C 10 s^−1^. Gene-specific primers for *clock1*, *bmal1*, *per1b*, *per2* and *dbp* have been previously described [10, 42]. We verified the efficiency of the primers by constructing standard curves for all genes investigated. Moreover, the dissociation curve was used to confirm the specificity of the amplicon. The relative levels of each sample were calculated by the 2^–ΔΔCT^ method (where CT is the cycle number at which the signal reaches the threshold of detection) [43]. As housekeeping genes we used *gapdh*, because of it is frequently used in zebrafish expression studies, and *loopern4*, an expressed repetitive elements recently showed as stable reference target for qPCR normalization [44]. Nearly identical results were observed with both housekeeping genes. Each CT value used for these calculations is the mean of three replicates of the same reaction.

### Data analysis

The videos were analysed by FishTracker, a software developed by the Computer Vision Research Group of the University of Murcia [40–41]. The program tracks the movement of the larvae and provides the spatial coordinates, corresponding to the X:Y position in the well. The distance between two consecutive points (X_1_:Y_1_; X_2_:Y_2_) was calculated using the distance formula derived from the Pythagoras´ theorem and data were arranged in 10 minutes batches for a total of 144 data per day. The locomotor activities were analysed using the chronobiology software “El Temps” (v. 275, Prof. Díez-Noguera, University of Barcelona) which allows actograms to be drawn and calculates the daily meanwave of the locomotor activity.

### Statistical analysis

All the results are expressed as means ± SEM. Data were normally distributed (D'Agostino-Pearson normality test, p<0.05) and all populations had the same variance (Bartlett's test for equal variances, p<0.05). One-way and two-way analysis of variance (ANOVA) were used to determine differences in the locomotor activity among ZT/CT, dpf and lighting conditions. Two-way ANOVA tests were also carried out to determine statistical differences in gene expression between ZT/CTs and lighting conditions. Tukey´s HSD post-hoc test was used for the multiple comparison among groups (p<0.05). ANOVAs were performed using SPSS 15.0 (SPSS Inc., Chicago, IL, USA). To evaluate the presence of a rhythmic gene expression over a defined period of 24 hours, Cosinor analyses were performed (El Temps, v. 275, Prof. A. Díez-Noguera, University of Barcelona, Spain).

The daily acrophase of the locomotor activity rhythm for each larvae was calculated and the average acrophase for each day and for each group was determined by vector addition. The Rayleigh test was used to test whether the acrophases deviated from uniform (p<0.05). Uniform scores test was applied to test for differences between the acrophases among days either intra- or inter-group (p<0.05) [45].

## Results

### Locomotor activity

**Fig 1:**
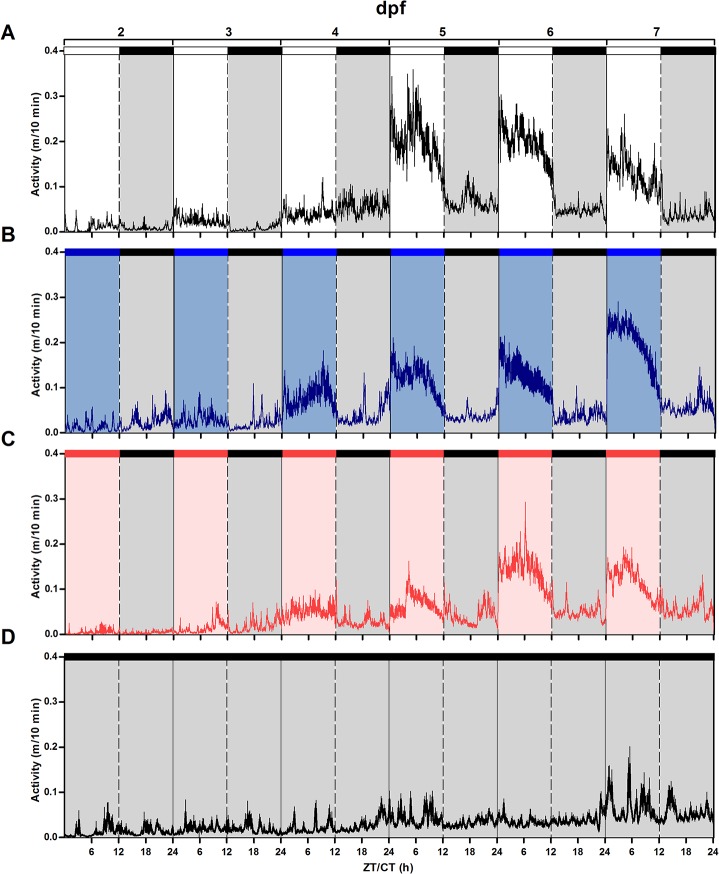
Mean waveforms of locomotor activity under different lighting conditions from 2 to 7 dpf. Larvae reared under different light conditions (**A**: LDW, n = 35, **B**: LDB, n = 30, **C**: LDR, n = 30, **D**: DD, n = 45). Vertical axis represents activity (m/10 min) and horizontal axis zeitgeber/circadian time (ZT/CT). Bars above each panel indicate the lighting conditions [black bars indicate darkness, white bars indicate white light (LDW), blue bars indicate blue light (LDB), and red bars indicate red light (LDR)] and the day post-fertilization (dpf). Data are expressed as mean ± SEM.

All larvae hatched between 2 and 3 dpf. At 4 dpf, larvae from LDB group started to display a daily rhythm of locomotor activity (Fig 1B; Cosinor, p<0.001). Daily rhythms of activity became significant at 5 dpf in LDW and LDR groups (Fig 1A and 1C; Cosinor, p<0.001 and p<0.01, respectively). Larvae reared under DD were arrhythmic during all the days recorded (Fig 1D; Cosinor, p>0.1). Larvae from all LD groups display the typical diurnal pattern of zebrafish, with higher activity (>65%) during the light phase.

**Fig 2:**
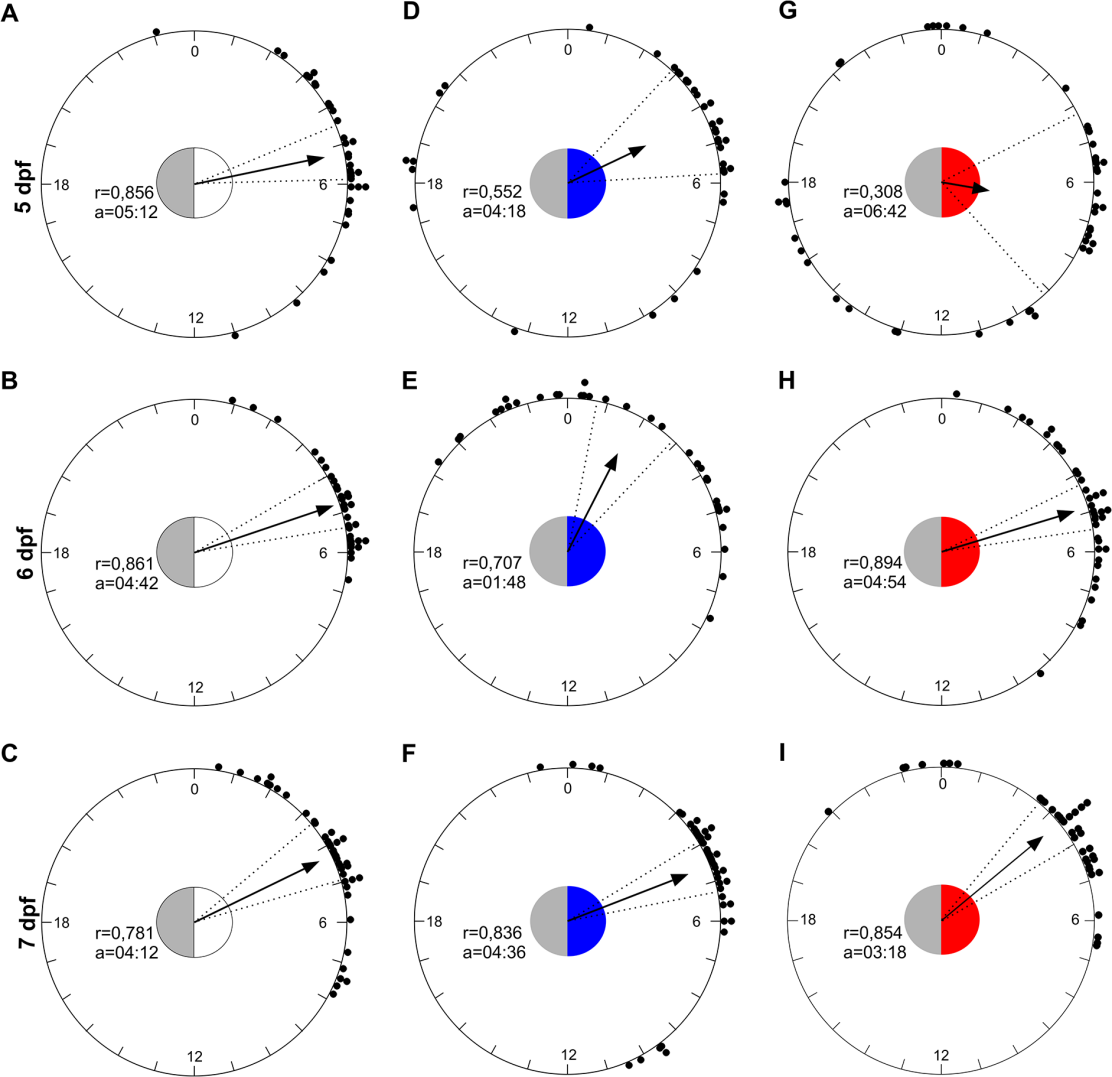
A circular representation of the phases of zebrafish activity across the 24 hours from 5 to 7 dpf under LDW, LDB and LDR. The dots represent the acrophase of each zebrafish larvae. The arrows indicate the average phases represented as vector and in each circle the mean vector length (r) and the mean acrophases (a) in ZT are reported. The circle inside each panel represents critical values of the Rayleigh test (p<0.05) and the coloured part show the duration of light phase (ZT 0–12). The dotted lines represent the confidence intervals.

To verify the accuracy of the entrained rhythm we estimated the time of acrophases respect to the lights on (ZT0) in all groups from 5 to 7 dpf. Using a circular statistic approach, we showed that the distribution of acrophases deviated from uniform in LDW, LDB and LDR groups (Fig 2; Rayleigh test, 0.05<p<0.001), and the mean acrophases fell between ZT 2 and 7 (Fig 2). The distribution of acrophases from 5 to 7 dpf differed only between LDB and LDR groups (Mardia-Watson-Wheeler Test: LDW: W_4_ = 5.4, p<0.05; LDB: W_4_ = 38.9, p<0.0001; LDR: W_4_ = 45.3, p<0.0001). The distribution among groups did not differ at 7 dpf (Mardia-Watson-Wheeler Test: W_4_ = 4.5, p>0.3), and the mean acrophases fell at ZT 03:18–04:36 (Fig 2).

**Fig 3:**
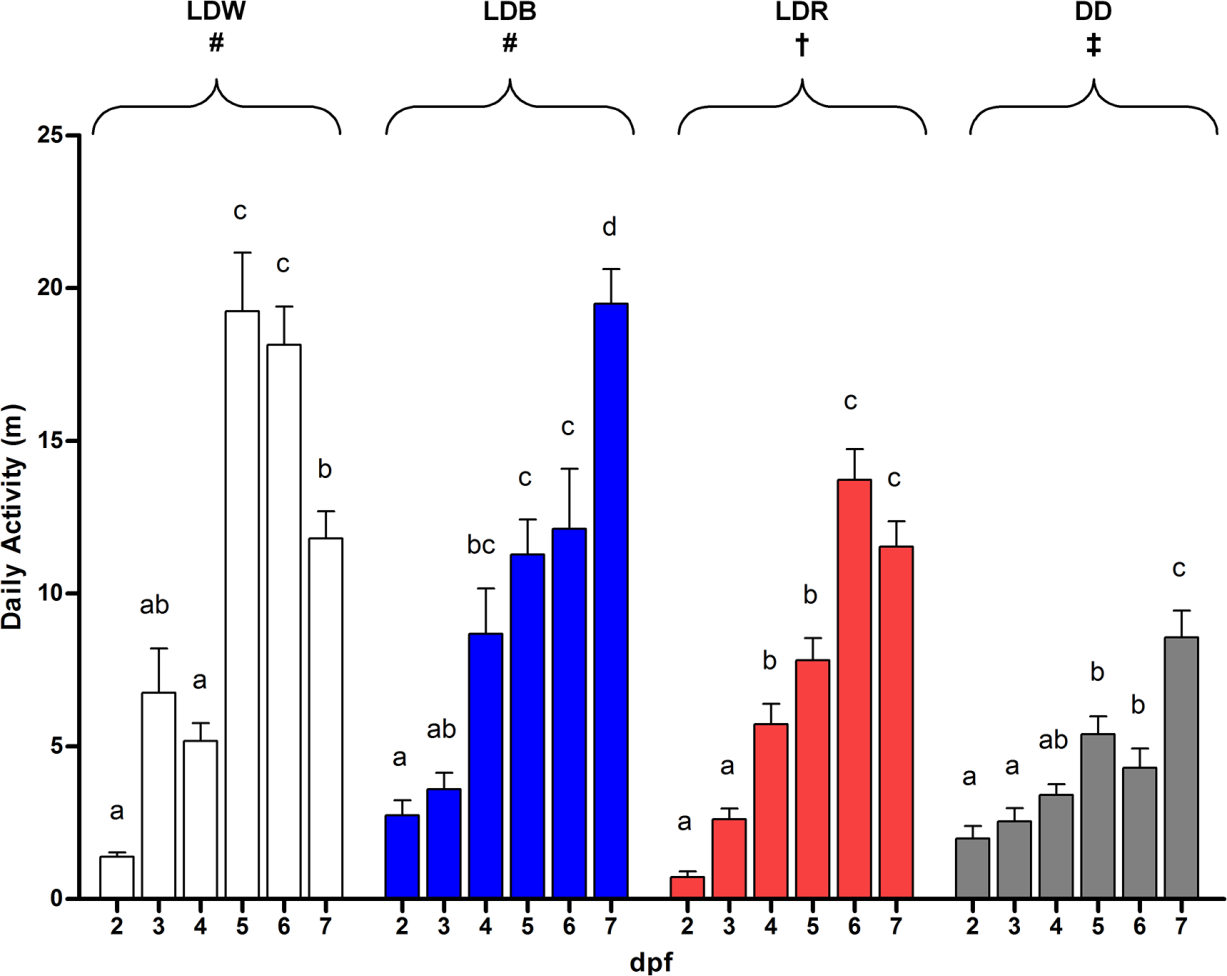
Daily activity under different lighting conditions from 2 to 7 dpf. Data are expressed as mean ± SEM. Letters indicate statistical differences between the different days for each lighting condition (one-way ANOVA; Tukey’s post-hoc test, p<0.05). Symbols indicate statistical differences between the lighting conditions (two-way ANOVA; Tukey’s post-hoc test, p<0.05).

All groups showed an increase in the total daily locomotor activity throughout development (two-way ANOVA, p<0.05) (Fig 3). Larvae kept under LDB and LDR showed an increase in the total daily activity from 4 dpf, whereas a significant increase in activity under LDW and DD occurred at 5 dpf (Figs 1 and 3). Considering the whole period of recording (5 days, from 2 to 7 dpf), larvae reared under LDW and LDB displayed significantly higher overall activity than larvae under LDR and DD (LDW: 60.17 m; LDB: 56.51 m; LDR: 42.05 m; two-way ANOVA, p<0.05). Furthermore, larvae reared in DD showed lower total activity with respect larvae reared in the other lighting conditions (DD: 27.36 m; two-way ANOVA, p<0.05).

### Clock gene expression

**Fig 4:**
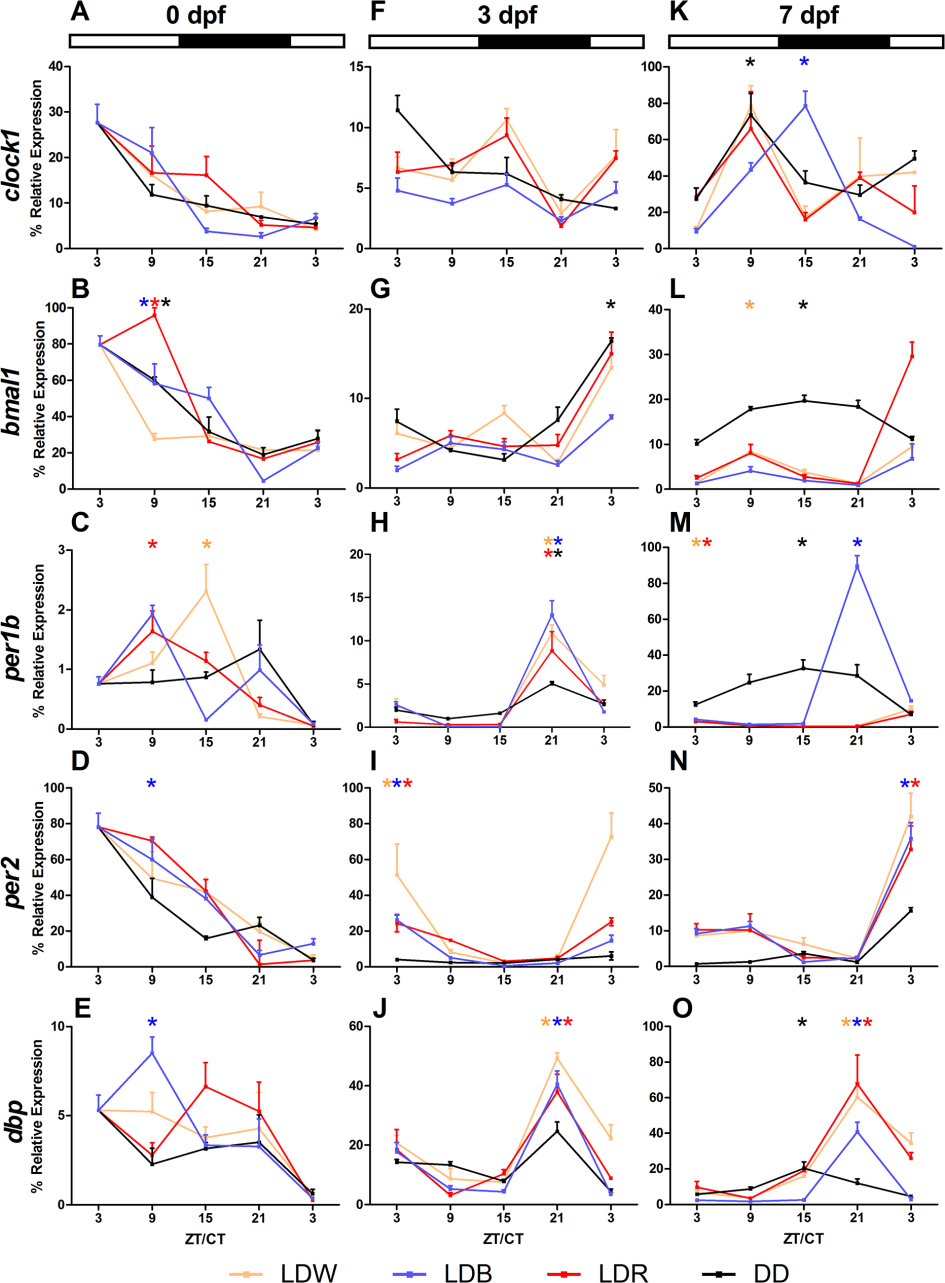
Daily expression levels of *clock1*, *bmal1*, *per1b*, *per2* and *dbp* at 0, 3, 7 dpf in zebrafish larvae. Larvae reared under different light conditions (LDW, LDB, LDR, DD) were sampled every 6 hours at 0 (**A**, **D, G, L, O**), 3 (**B**, **E, H, M, P**) and 7 (**C**, **F, I, N, Q**) dpf. Data are expressed in % (100% is the maximum level detected for each gene in all dpf) and each value represents mean ± SEM (n = 4). The bars above each group indicate the daily LD cycle. White bars represent the light phase and black bars represent phases of darkness. The DD group was kept under constant darkness. Asterisks indicate significant rhythms identified by Cosinor analysis (p<0.05).

Embryos during the first 24 hours of life (0 dpf) showed variation of expression levels in all genes investigated (one-way ANOVA, p<0.05; Fig 4A–4E) and these variations were not affected by lighting conditions (two-way ANOVA, p>0.3; Fig 4A–4E). For the positive loop of the molecular clock, Cosinor analysis showed a significant rhythmicity (p<0.05) for *bmal1* under LDW, LDR and DD, whereas *clock1* expression levels were in all lighting conditions arrhythmic (p >0.05, Fig 4A and 4B). For the negative elements, *per1b* and *per2* rhythmicity appeared only in some lighting conditions (Cosinor, p<0.05; *per1b*: LDW and LDR; *per2*: LDB; Fig 4C and 4D). The clock-controlled gene *dbp* was rhythmic in LDB, but not under the other lighting conditions (Fig 4E). At 0 dpf all rhythmic genes displayed their acrophases during the light phase, with the exception of *per1b* under LDW (ZT 13:13 h) (Table 1).

**Table 1:** Mesor, Amplitude and Acrophase defining clock gene expression rhythms at 0, 3 and 7 dpf.

		0 dpf			3 dpf			7 dpf		
		Mesor (r.e.)	Amplitude (r.e.)	Acrophase ZT/CT	Mesor (r.e.)	Amplitude (r.e.)	Acrophase ZT/CT	Mesor (r.e.)	Amplitude (r.e.)	Acrophase ZT/CT
***clock1***	LDW	-	-	-	-	-	-	-	-	-
	LDB	-	-	-	-	-	-	7.5	7.82	13:21
	LDR	-	-	-	-	-	-	-	-	-
	DD	-	-	-	-	-	-	0.65	0.32	09:04
***bmal1***	LDW	-	-	-	-	-	-	0.61	0.55	08:33
	LDB	5.81	4.13	07:19	-	-	-	-	-	-
	LDR	6.56	5.37	07:36	-	-	-	-	-	-
	DD	5.95	3.9	06:35	0.95	0.6	01:18	2.35	0.71	15:07
***per1b***	LDW	1.31	1.17	13:13	4.47	6.54	22:07	2.83	4.29	02:32
	LDB	-	-	-	4.44	7.39	21:05	29.6	53.89	21:03
	LDR	1.07	0.92	11:03	3.08	5.24	21:04	2.17	3.11	03:12
	DD	-	-	-	3.13	2.48	20:34	28.8	15.2	15:21
***per2***	LDW	-	-	-	2.73	4.39	03:06	-	-	-
	LDB	5.27	4.58	08:26	0.93	1.43	03:24	1.29	1.54	04:22
	LDR	-	-	-	1.5	1.58	04:24	1.15	1.28	04:24
	DD	-	-	-	-	-	-	-	-	-
***dbp***	LDW	-	-	-	7.45	8.11	21:33	10.1	10.94	20:28
	LDB	1.61	1.13	09:58	5.6	6.85	21:13	4.79	7.77	20:04
	LDR	-	-	-	6.23	6.76	21:09	10.27	12.67	20:01
	DD	-	-	-	-	-	-	4.56	2.9	15:32

Mesor and Amplitude are given as relative expression values (r.e.) and Acrophase as ZT. Rhythms are considered significant when p<0.05. Only statistically significant values (p<0.05) are reported.

At 3 dpf larvae had hatched, but the swim bladder was not developed and the yolk sac was the only source of energy. At this developmental stage, the negative element *per1b* was rhythmically expressed (one-way ANOVA, p<0.001; Cosinor, p<0.005; Fig 4H; Table 1), and its expression was mainly affected by LDB and DD conditions (two-way ANOVA, p<0.01; Fig 4H). *Per2*, other negative element of the loop and a light-inducible gene, also showed a significant rhythmic variation of the expression levels across the day under all wavelengths (one-way ANOVA, p<0.001; Cosinor, p<0.04; Fig 4I; Table 1), but not in DD (one-way ANOVA, p>0.09; Fig 4I). The highest induction of *per2* by light was under LDW (two-way ANOVA, p<0.001; Fig 4I). *Per1b* and *per2* rhythms were not in phase: *per1b* showed the acrophase ranging between ZT/CT 20:34–22:07, *per2* at ZT/CT 3:06–4:24 (Table 1). Although ANOVAs revealed a significant variation during the 3 dpf (one-way ANOVA, p<0.05) and an effect of lighting conditions (two-way ANOVA, p<0.03), both positive elements *clock1* and *bmal1* did not show rhythmic variations of expression levels (Cosinor, p>0.1; Fig 4F; Table 1), except for *bmal1* under DD (Cosinor, p<0.02; Fig 4G; Table 1). *Dbp* was rhythmic (one-way ANOVA, p<0.003; Cosinor, p<0.001; Fig 4J; Table 1) and affected by lighting conditions (two-way ANOVA, p<0.001). *Dbp* rhythms had acrophases between 21:09–21:33 ZT/CT (Fig 4J; Table 1).

At 7 dpf larvae started exogenous feeding and were able to swim freely. In contrast to the other developmental stages analysed, *clock1* expression levels at 7 dpf showed daily variations during the day (one-way ANOVA, p<0.04) and oscillated rhythmically under LDB and DD (Cosinor, p<0.008) with acrophases at ZT 13:21 and CT 09:04, respectively (Fig 4K). The other positive element investigated, *bmal1*, showed a similar daily pattern: rhythmic in DD and LDW (one-way ANOVA, p<0.04; Cosinor, p<0.03; Fig 4L; Table 1) with acrophases at CT 15:07 and ZT 8:33, respectively. The negative elements *per1b* and *per2* showed temporal variation in expression levels (one-way ANOVA, p<0.002). *Per1b* was rhythmic under all lighting conditions (Cosinor. p<0.01, Table 1, Fig 4M) with acrophases at ZT 2:32–3:12 under LDW and LDR respectively and ZT/CT 15:21–21:03 under DD and LDB, respectively. *Per2* was rhythmic only under LDB and LDR (Cosinor, p<0.01, Table 1) with acrophases at ZT 4:24 under LDB and 4:24 under LDR (Fig 4N). *Per1b* and *Per2* rhythms were strongly influenced by lighting conditions (two-way ANOVA, p<0.001). *Dbp* expression levels changed during the 7 dpf (one-way ANOVA, p<0.001) and showed rhythmic oscillations depending on the lighting conditions (two-way ANOVA, p<0.001). *Dbp* expression showed acrophase during the dark phase: ZT 20:28 under LDW, 20:04 under LDB and 20:01 LDB and CT 15:32 under DD (Cosinor, p<0.003; Fig 4O; Table 1).

**Fig 5:**
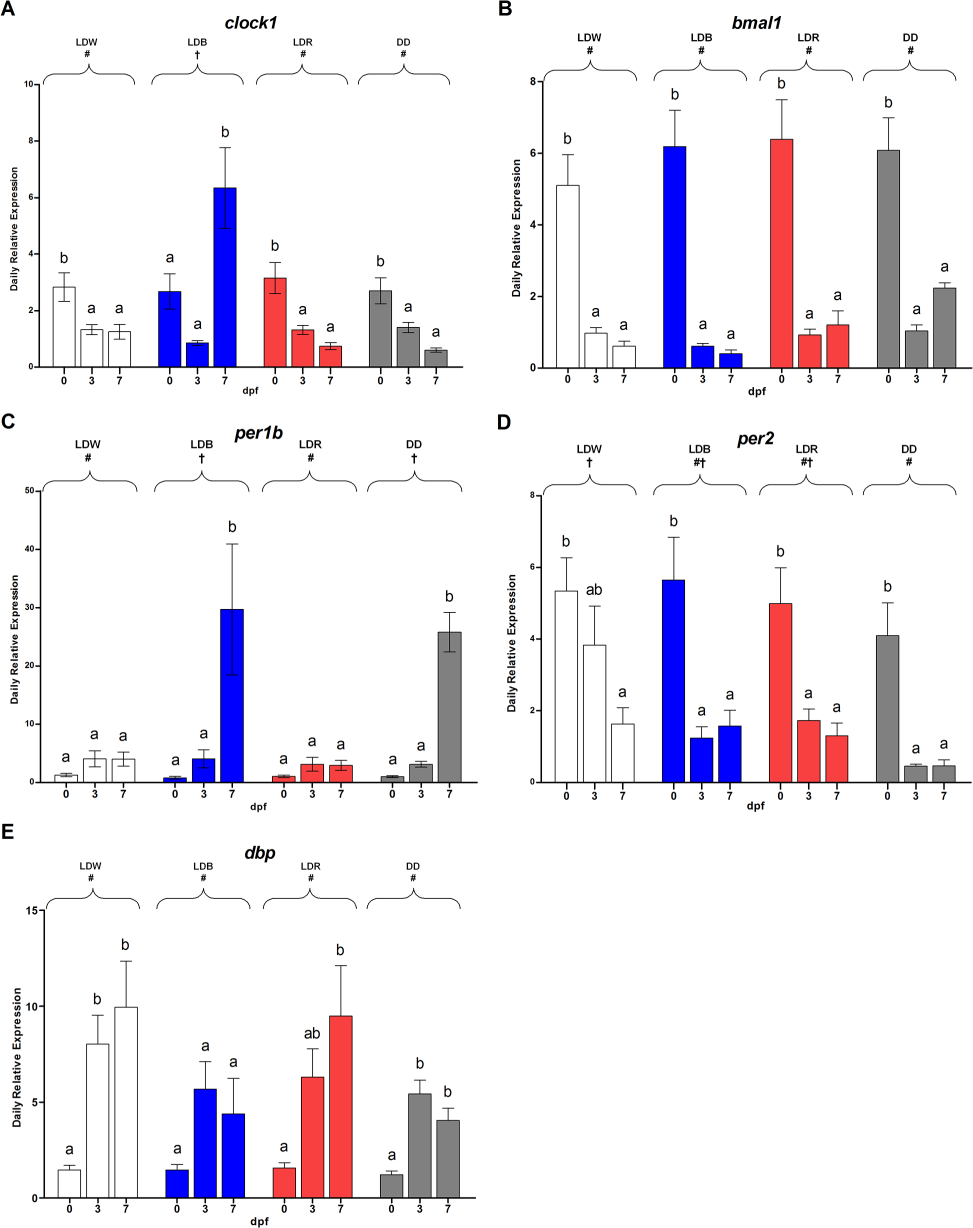
Daily expression levels at 0, 3, 7 dpf of *clock1 bmal1*, *per1b*, *per2 and dbp* under different lighting conditions. The data are expressed as mean ± SEM. The letters above each bar indicate significant differences for each condition among the days (one-way-ANOVA, p<0.05). The symbols on the top indicates statistically differences between the lighting conditions (two-way-ANOVA, Tukey’s post-hoc test, p<0.05).

The comparison of mean expression levels of each gene showed differences depending on the experimental conditions (Fig 5). For instance, the highest levels of *clock1*, *bmal1* and *per2* expression levels were at 0 dpf (one-way ANOVA, p<0.01; Fig 5A, 5B and 5D). Differently, *per1b* and *dbp* showing the highest values at 3 and 7 dpf (one-way ANOVA, p<0.05; Fig 5C and 5E). The daily mean expression level of *dbp* was higher at 3 and 7 dpf respect to 0 dpf in LDW, LDR and DD (two-way ANOVA, p<0.001; Fig 5E), whereas in LDB the mean levels did not change during the first week of life (Tukey’s post-hoc, p<0.05; Fig 5E). *Clock1* relative expression was mainly influenced by LDB (Tukey’s post-hoc, p<0.008, Fig 5A). No statistical differences were detectable for both *bmal* and *dbp* among the lighting conditions (two-way ANOVA, p<0.2, Fig 5B and 5E). LDB and DD influenced the *per1b* mean expression differently from LDW and LDR (Tukey’s post-hoc, p<0.03; Fig 5C). The effect LDW on *Per2* expression is different from DD but not from LDB and LDR (Tukey’s post-hoc, LDW vs DD: p<0.01, LDW vs LDB-LDR: p<0.6, LDB-LDR vs DD: p<0.3; Fig 5D).

## Discussion

Solar light is a complex environmental signal that influences the evolution of most biological processes on the Earth. Light is characterized by daily changes in irradiance, wavelength composition, direction and polarization [46]. In the last years many investigations are taking into account a significant role of the different wavelengths [20–22]. Here we found that light wavelength affected the larval behaviour and the onset of the clock gene rhythmicity. At the behavioural level, larvae were arrhythmic under DD, while they developed daily activity rhythms under LD cycles, which appeared earlier in LDB (4 dpf) than in LDW or LDR (5 dpf). Furthermore, larvae reared under LDW and LDB displayed significantly higher overall activity than larvae under LDR. At 7 dpf the phase of the rhythm in all lighting conditions is identical and the acrophases fell in the early day (ZT 3–4). Previous investigations showed that the rise of swimming behaviour in zebrafish larvae is linked to the maturation of serotoninergic neurons [47]. We cannot exclude that the anticipation of 1 day (4 dpf respect to 5 dpf) in the onset of the daily rhythm of locomotor activity and the high overall activity under blue light depends to a stimulation of the serotoninergic system by this lighting conditions.

Previous studies have shown that zebrafish behaviour under constant lighting conditions is regulated by an endogenous clock and that the LD cycle sets the phase of this clock [7, 16]. For instance, LD cycles are required for the correct onset of behavioural rhythmicity in zebrafish larvae [16]. The amplitude of activity rhythms is directly correlated with the number of LD cycles to which embryos are subjected before they are transferred to DD conditions. Subjecting the zebrafish embryos to only one or two LD cycles after fertilization has been seen to significantly reduce the number of animals displaying circadian rhythmicity [16]. Our results agree with this study, since larvae reared under DD conditions, which only received 3 hours of light after fertilization, did not develop locomotor rhythmicity and displayed lower activity levels than fish reared under LD cycles. A recent investigation also showed the effects of DD on zebrafish larval development, growth and survival [20]. Zebrafish larvae raised in DD died before 18 days post-hatching (dph). Interestingly, larvae transferred to an LD cycle at 5 and 10 dph showed an improved survival rate compared with the larvae maintained in DD [20], which further underlines the importance of LD cycles to sustain normal development during early larval stages.

The analysis of clock gene ontogeny revealed different results in the animals under DD and LD conditions. For instance, under DD *per2* rhythms failed to establish during the development, which has been observed in other fish species such as the medaka (*Oryzias latipes*) and the Senegalese sole (*S*. *senegalensis*) [15, 48–49]. This effect of DD conditions would be explained by the fact that *per2* is a light-inducible gene, and thus requires the presence of light for its daily rhythmicity to develop correctly [50]. Interestingly, the presence of constant light does not make for the regular expression of clock genes. In the rainbow trout, *Oncorhynchus mykiss*, the clock genes *per1* and *clock* showed persistent rhythmicity in the larvae reared under LD conditions from 0 to 58 dpf, but not under constant lighting, when the rhythmicity is lost or developed later respect to the LD conditions [51]. The other negative element investigated, *per1b*, showed rhythmicity in DD from 3 dpf, whereas *clock1* and *dbp* were rhythmic from 7 dpf. Only the positive element *bmal1* had a rhythmic expression under DD from the embryo stage (0 dpf).

After 3 days of exposition to LD cycles, *per1b*, *per2* and *dbp* showed a significant variation in the daily gene expression. On the contrary, genes from the positive loop of the clock, *clock1* and *bmal1*, needed a longer time to start oscillating and after 7 days in LD cycles and only in two conditions they were rhythmic (*clock1* in LDB and *bmal1* in LDW). These discrepancies among the times of occurrence of rhythmicity among key components of the circadian clock has been suggested in other studies in fish [15, 48–49].

Interestingly, we observed a daily rhythm at 0 dpf under all lighting conditions for some genes, with the acrophase located during the first hours after fertilization and expression levels falling during the rest of the day. A similar response has been observed previously in zebrafish and in Senegalese sole [48, 52]. Rather than a real rhythmicity driven by an endogenous clock, this result might depend on a direct light induction of the first hours of light after fertilization [52], since in the present experiment even embryos reared in DD received 3 hours of light, while eggs were being collected, or alternatively, to the presence of maternal RNA (*clock1*, *bmal1* and *per2* at ZT 3 of 0 dpf) [53].

Clock genes from the negative loop (*per1b* and *per2*) displayed rhythmic expression early during development compared with *clock1* and *bmal1*, results that did not depend on the photoreceptive system in zebrafish larvae. By 5 dpf, the larval retina is differentiated and functional, displaying responses evoked by visual stimuli. It expresses different opsins including melanopsin, a photopigment involved in circadian photoreception. Recent investigation in *Danio rerio* ZEM-2S cells points to melanopsin as the photopigment that mediating the photoresponse increasing *per2* and *cry1a* and slightly modulating *per1b* and *cry1b* expression [54–56]. The pineal gland is formed around 18–22 hours post-fertilization and starts displaying rhythmicity under LD cycles at 1 dpf [30, 57]. Photoreceptive cells expressing TMT-opsin and melanopsin are present in the brain of 3–6 day old zebrafish larvae [58–59]. To date, it is unknown which requirements or processes are involved in delaying the appearance of *clock1* and *bmal1* rhythms compared with *per*s. However, in medaka too, the rhythmic expression of *per* is detected very early during development and rhythmic *clock* and *bmal* expression occurs later [49].

In the case of light wavelength, its effects on the ontogeny of fish are scarcely understood to date. Recent papers on this topic have focused on wavelength effects on larval performance, survival and the occurrence of malformations, finding that, in general, short (blue) wavelengths are better for fish development than long (red) wavelengths [20, 22]. A drastic effect of short wavelengths on larval behaviour has also been found in the Senegalese sole [60]. The blue light condition was able to generate a switch in locomotor activity, changing the active phase from diurnal to nocturnal during larval metamorphosis onset. Conversely, long (red) wavelengths did not show any effect on the locomotor activity [60]. Also zebrafish PAC-2 cells seem to be more sensitive to short wavelengths: the expression level of light-inducible genes, *cry1a*, *cry5* and *per2*, was higher when submitted to blue than to red lighting conditions [61]. Our results agree with those obtained in the other fish species reported above, since both behavioural and clock gene rhythms appeared earlier in the larvae reared under short wavelengths (LDB) than in those reared under long wavelengths (LDR).

In summary, our study provides novel insights into the ontogeny and the effects of lighting conditions on molecular daily rhythms, and how these rhythms are reflected in the behavioural rhythmicity of zebrafish larvae. The present results also underline the relevance of lighting conditions on fish development. The LD cycle and specific wavelength is essential for normal development of the circadian system. These conditions should be carefully considered when fish embryos and larvae of zebrafish or other model fish species reared in laboratory facilities and in fish hatcheries for aquaculture companies.
